# Substance-related poisoning hospitalizations and homelessness in Canada: a descriptive study

**DOI:** 10.24095/hpcdp.44.5.02

**Published:** 2024-05

**Authors:** Rebecca Plouffe, Rochelle White, Heather Orpana, Vera Grywacheski

**Affiliations:** 1 Centre for Surveillance and Applied Research, Health Promotion and Chronic Disease Prevention Branch, Public Health Agency of Canada, Ottawa, Ontario, Canada; 2 Cancer Screening Analytics, Clinical Institutes and Quality Programs, Ontario Health, Toronto, Ontario, Canada; 3 School of Psychology, University of Ottawa, Ottawa, Ontario, Canada

**Keywords:** opioids, overdose, fentanyl, housing, mental disorder, hospitalization

## Abstract

**Introduction:**

The objective of this analysis is to describe patient demographics, the context, characteristics and outcomes of a substance-related poisoning, and the recorded mental disorder of people with housing and those experiencing homelessness.

**Methods:**

Hospitalization data for Canada (except Quebec) from 1 April 2019 to 31March 2020 were retrieved from the Canadian Institute for Health Information (CIHI) Discharge Abstract Database using ICD-10-CA codes for up to 25 diagnoses for substance-related poisonings, homelessness status and other characteristics relevant to the patient’s hospitalization. We compared the characteristics of people experiencing homelessness with those of people who were housed, and their substance-related poisoning hospitalizations, using chi-square, t tests and Fisher exact test.

**Results:**

There was a higher proportion of males, younger individuals and people with recorded mental disorders among people experiencing homelessness hospitalized for a substance-related poisoning than among their housed counterparts. Substance-related poisonings among people experiencing homelessness were more likely to be accidental, involve opioids and stimulants (most frequently fentanyl and its analogues and heroin), result in lengthier hospitalizations and end with leaving the hospital against medical advice.

**Conclusion:**

These findings can be used to strengthen strategies and interventions to reduce substance-related harms in priority populations, particularly those experiencing homelessness.

**Highlights**

- People who are homeless were vastly overrepresented among people hospitalized for substancerelated poisonings.
- In fiscal year 2019/2020, people experiencing homelessness who were hospitalized for substancerelated poisonings spent, on average, about 4 days longer in hospital than people with housing.
- Almost one-quarter (23%) of the hospitalizations of people experiencing homelessness ended with the patients leaving against medical advice, compared to 8% of hospitalizations for people with housing.
- An important area for future research would be to identify ways in which hospitals can retain and treat this at-risk population.
- Research can also help inform additional prevention and harm reduction activities.

## Introduction

Canada continues to experience an overdose crisis, with substance-related morbidity and mortality increasing significantly since 2016.^1^ Between January 2016 and December 2020, there were 24 671 opioid-related and 11 176 stimulant-related poisoning hospitalizations in Canada (excluding Quebec).^1^ Although most regions of the country have been affected, British Columbia, Alberta and Ontario continue to have the most opioid and stimulant-related poisoning hospitalizations.^1^ Some subpopulations appear to be disproportionately affected by the overdose crisis, including people experiencing homelessness and housing insecurity.^2^

The rates of substance use are disproportionally high among people experiencing homelessness, and they are at a greater risk of substance-related harms compared to people with housing.^3^-^7^ People who are homeless are also more likely than people with housing to be diagnosed with a mental health disorder, remain hospitalized for longer, and be readmitted within 30 days following discharge.^4^-^9^

On average, at least 235000 people experience homelessness in a given year in Canada, and at least 35000 on a given night.^2^ Across the country, an additional 50000 people could be experiencing hidden homelessness every night, that is, staying temporarily with friends, relatives or others because they have no other housing option and no immediate prospect of permanent housing.^2^ The number of people experiencing homelessness in Canada is very difficult to estimate, but it is thought to be increasing, possibly also as a result of job losses and evictions during and since the COVID-19 pandemic.^2^,^10^,^11^

The objective of this analysis is to describe patterns of substance-related poisoning hospitalizations in Canada (excluding Quebec) among people with housing and people experiencing homelessness, using the Canadian Institute for Health Information (CIHI) Discharge Abstract Database (DAD) during the pre-pandemic year of 1 April 2019 to 31 March 2020. This study also examines patterns by patient demographics (sex and age); context of the poisoning (substances involved and intention of the poisoning); hospitalization characteristics and outcomes (length of stay, intensive care unit admission and discharge disposition); and recorded mental disorders.

To our knowledge, this is the first study comparing the characteristics of those experiencing homelessness and those who are housed among people hospitalized for a substance-related poisoning across Canada using this data source. The results of this study can be used to better understand the intersection of homelessness, mental health and substance-related harms and how hospital care is experienced differently by people who are homeless.

## Methods

***Data source***

We obtained data from the DAD, which captures acute inpatient discharge records for hospitalizations across Canada, excluding Quebec. In 2019–2020, the DAD had full coverage for acute inpatient care, except from one facility that did not submit data for six periods (an estimated total of 1100 missing abstracts).^12^ Data were presented for the time from 1 April 2019 to 31 March 2020. *International Classification of Diseases and Related Health Problems, Tenth Revision, Canada* (ICD-10-CA) codes were used to capture up to 25 diagnoses from the patient’s hospitalization.

***Identifying the study sample***

The methodology used to identify substance-related poisonings was adapted from existing CIHI methods.^13^,^14^ Trained coders reviewed medical records and assigned substance specific ICD-10-CA codes according to CIHI coding directives.^13^,^15^ Substance-related poisonings may be recorded in a patient’s chart based on toxicological analyses, self-report and/or responsiveness to treatment received (for instance, reversal of an opioid poisoning after being administered naloxone). Poisonings of interest were included if they were due to the following substances: opioids (T40.0, T40.1, T40.2, T40.20–T40.23, T40.28, T40.3, T40.4, T40.40, T40.41, T40.48, T40.6); stimulants (T40.5, T43.6); cannabis (T40.7); hallucinogens (T40.8, T40.9); alcohol (T51); other depressants (T42.3, T42.4, T42.6, T42.7); and psychotropic drugs (T43.8, T43.9).

This analysis was limited to significant poisonings, defined as cases where the poisoning influenced the duration of the time the patient spent in hospital and the treatment they received. Secondary diagnoses and unconfirmed or query diagnoses were excluded.

***Additional variables***

**Homelessness status**

Any mention of the ICD-10-CA code Z59.0 on a patient discharge abstract was used to note confirmed or unconfirmed and suspected instances of homelessness status. Homelessness status upon admission to hospital is mandatory to code when mentioned in physician documentation or noted on routine review of the medical record.

**Intention of poisoning**

Intention of the poisoning was identified in line with CIHI coding standards, where coders assign an external cause ICD-10-CA code indicating whether the poisoning was accidental (X41, X42, X45), intentional (X61, X62, X65) or undetermined (Y11, Y12, Y15). Confirmed and suspected diagnoses were included in the intention analysis. Records containing one or more poisonings with a missing associated external cause code were excluded from analyses of intention.

**Recorded mental disorders**

Consistent with CIHI methodology, recorded mental disorders were identified using any relevant ICD-10-CA diagnoses recorded on the patient discharge abstract during their stay for the substance-related poisoning.^15^,^16^ It is mandatory to record the diagnosis of a mental disorder if having this disorder significantly affects the treatment received, requires treatment beyond maintenance of the pre-existing disorder or increases the length of stay in hospital by at least 24 hours.

All ICD-10-CA codes for a mental disorder on the patient discharge abstract were captured, including confirmed and suspected diagnoses. The following were included: substance-related and addictive disorders (F10–F19, F55, F63.0); schizophrenia and other psychotic disorders (F20–F25, F28, F29); mood disorders (F30–F34, F38, F39, F53.0, F53.1); anxiety disorders (F40, F41, F93.0–F93.2, F94.0); selected disorders of personality and behaviour (F60–F62, F68 [excluding F68.1], F69); and other mental disorders (F42–F45, F48.0, F48.1, F48.8, F48.9, F50–F52, F53.8, F53.9, F54, F59, F63 [excluding F63.0], F68.1, F90–F92, F93.3, F93.8, F93.9, F94.1, F94.2, F94.8, F94.9, F95, F98.0, F98.1–F98.5, F98.8, F98.9, F99, O99.3). Some examples of “other mental disorders” covered by these ICD-10-CA codes include hypochondriacal disorder, eating disorders, nonorganic sleep disorders, conduct disorders, and posttraumatic stress disorder.

**Length of stay in hospital and discharge disposition**

Total length of stay in hospital was calculated as the sum of the number of days a patient was in acute inpatient care and alternate level of care. Acute inpatient care length of stay describes when a patient is receiving necessary treatment for a disease or severe episode of illness for a short period; alternate level of care describes when a patient is occupying a bed, but not requiring the intensity of services provided in that care setting.

Discharge disposition refers to the status of the patient upon discharge or where the patient is discharged to, and is identified by examining the patient’s hospitalization record.

***Analysis***

We conducted descriptive analyses of substance-related poisoning hospitalizations among people experiencing homelessness as well as among housed people (in order to have a reference category). Percentages of substance-related poisoning hospitalizations with a specific recorded mental disorder were calculated based on the denominator of the total study population; these may exceed 100% when summed because of polysubstance poisonings and diagnoses of multiple mental disorders. Counts of less than five per disaggregated category were suppressed in accordance with the CIHI privacy policy.^17^

We used a Pearson chi-square test to determine significant associations between housing status and categorical variables, and a Fisher exact test when expected counts for cells were less than five. A Satterthwaite *t* test was used to test differences by housing status for continuous variables. 

All analyses were completed using statistical package SAS Enterprise Guide version 7.1 (SAS Institute Inc., Cary, NC, US).

## Results

Between April 2019 and March 2020, there were 10659 substance-related poisoning hospitalizations in Canada (excluding Quebec). Approximately 6% (623) of these were recorded among people experiencing homelessness.

***Patient demographics***

**Figure 1:**
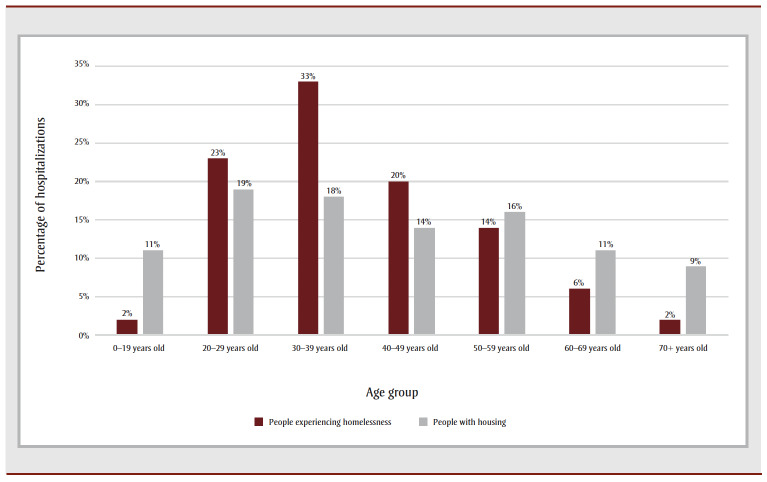
Age groups of patients hospitalized for a substance-related poisoning, by people experiencing homelessness and people with housing, Canada (excluding Quebec), April 2019 to March 2020

Among those hospitalized for substance-related poisonings, there was a higher proportion of males experiencing homelessness (71%) than females (29%), while among those with housing, slightly more females (53%) than males (47%) were hospitalized (Table 1). Of those hospitalized for substance-related poisoning, the mean age of people experiencing homelessness was lower than the mean age of their housed counterparts (39.2 vs. 42.5 years; *p* < 0.001) (Figure 1).

**Table 1:** Demographics of patients hospitalized for a substance-related poisoning, by people experiencing homelessness and people with housing, Canada (excluding Quebec), April 2019 to March 2020

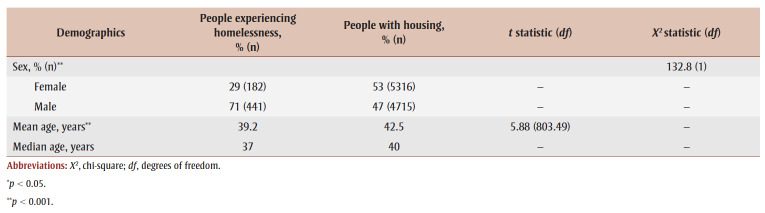

***Hospitalization characteristics and outcomes***

People who were homeless stayed for a significantly longer time in hospital for a substance-related poisoning than those with housing (11.0 vs. 6.6 days; *p*<0.05) (Table 2). The proportions of hospitalizations admitted into intensive care did not differ between the two population groups, but people experiencing homelessness had a higher mean length of stay in alternate level of care than those with housing (3.7 vs. 0.8 days; *p* < 0.05). Among individuals with housing, 8% discharged themselves from the hospital against medical advice, whereas 23% of individuals who were homeless did the same (*p* < 0.001). There was no difference between the two population groups in the proportions who died while hospitalized for a substance-related poisoning.

**Table 2:** Substance-related poisoning hospitalization characteristics and outcomes among people experiencing homelessness and people with housing, Canada (excluding Quebec), April 2019 to March 2020

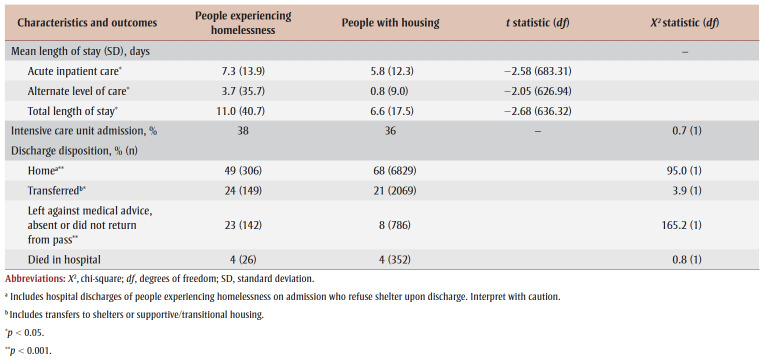

The majority (68%) of people with housing who were hospitalized for substance-related poisoning were discharged home. In comparison, 49% of hospitalizations of people experiencing homelessness on admission and who refused shelter upon discharge were “discharged home,” suggesting that this finding should be interpreted with caution.

***Substances involved in poisoning hospitalization***

Opioids were the most common type of substance involved in hospitalizations for a substance-related poisoning (Table 3), but to a greater extent among people experiencing homelessness than among people with housing (61% vs. 40%; *p* < 0.001). Stimulants, such as cocaine and methamphetamine, were also involved in a greater proportion of hospitalizations of people who were homeless (29%) compared to people with housing (29% vs. 19%; *p*<0.001). In contrast, other depressants, for example, benzodiazepines and other sedatives, were more common in hospitalizations of people with housing compared to those experiencing homelessness (39% vs. 19%; *p* < 0.001).

**Table 3:** Substances involved in hospitalizations for a substance-related poisoning of people experiencing homelessness and of people with housing, Canada (excluding Quebec), April 2019 to March 2020

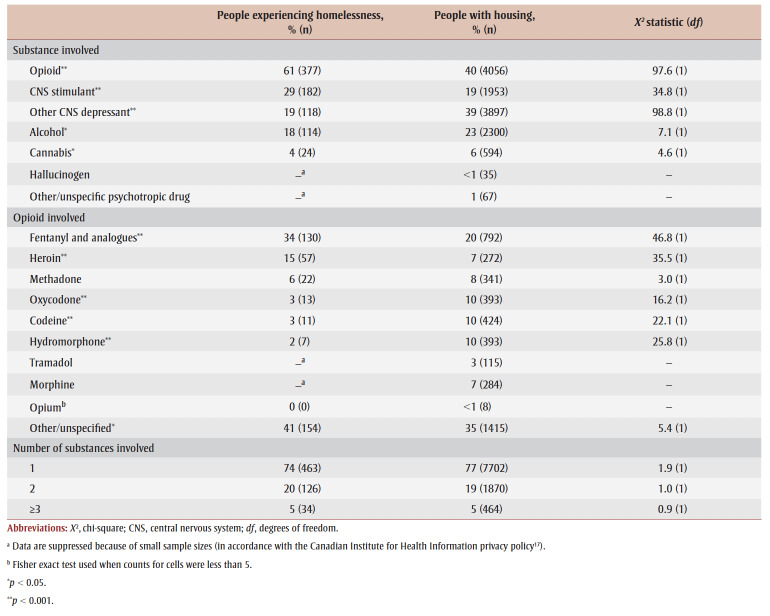

Where an opioid was involved in a poisoning hospitalization, fentanyl and its analogues (34% vs. 20%; *p* < 0.001) and heroin (15% vs. 7%; *p* < 0.001) were more prevalent in higher proportions of people experiencing homelessness than of people with housing. In contrast, oxycodone, codeine and hydromorphone were significantly more prevalent in hospitalizations of people with housing.

The percentage of substance-related poisoning hospitalizations that involved one, two or three or more substances did not differ by housing status.

***Intention of the poisoning***

Higher proportions of substance-related poisoning hospitalizations were recorded as accidental among people who were homeless than among people with housing (62% vs. 45%; *p* < 0.001) (Table 4). People with housing had a higher proportion of such hospitalizations recorded as intentional self-harm (46% vs. 26% for people experiencing homelessness; *p*<0.001). This pattern was also observed among females and males separately, although the magnitude of the differences varied.

**Table 4:** Intention of poisoning among hospitalizations for a substance-related poisoning of people experiencing homelessness and of people with housing, Canada (excluding Quebec), April 2019 to March 2020

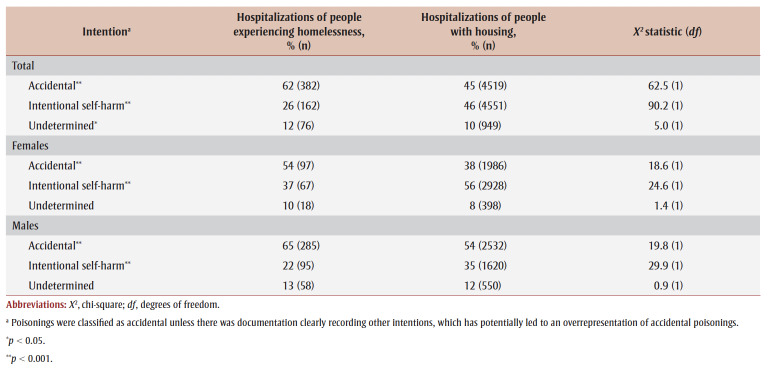

***Recorded mental disorders***

People experiencing homelessness who were hospitalized for substance-related poisonings had a higher proportion of mental disorders recorded during their hospital stay than those with housing (61% vs. 52%; *p* < 0.001) (Table 5). The most commonly recorded mental disorders for both populations were substance-related and addictive disorders, although a significantly greater proportion of people who were homeless than people with housing had this diagnosis (51% vs. 25%; *p* < 0.001). People with housing who were hospitalized for substance-related poisonings had a higher proportion of recorded mood disorders (21% vs. 11%; *p* < 0.001) and of recorded anxiety disorders (9% vs. 3%; *p* < 0.001) than their counterparts who were experiencing homelessness.

**Table 5:** Mental disorder recorded during hospitalizations for substance-related poisoning of people experiencing homelessness and of people with housing, Canada (excluding Quebec), April 2019 to March 2020

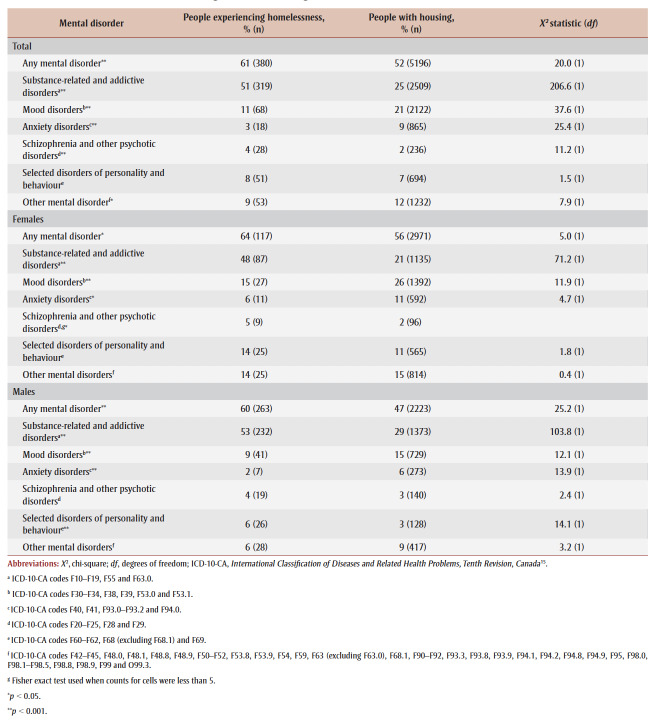

Stratification by sex showed significant differences in the distribution of substance-related poisoning hospitalizations with various mental disorders. Females experiencing homelessness were significantly more likely to have substance-related and addictive disorders (48% vs. 21%; *p* < 0.001) and schizophrenia and other psychotic disorders recorded (5% vs. 2%; *p* < 0.05) compared to their housed counterparts. Conversely, housed females were more likely to have mood (26% vs. 15%; *p* < 0.001) and anxiety disorders recorded (11% vs. 6%; *p*<0.05) compared to females who were homeless.

A similar trend was observed among males, with the most substantial difference between the two populations in diagnosed substance-related and addictive disorders. Among males experiencing homelessness, 53% had such a diagnosis compared to 29% of housed males (*p*< 0.001). Housed males were more likely to have mood disorders (15% vs. 9%; *p*<0.001) and anxiety disorders recorded (6% vs. 2%; *p* < 0.001) and less likely to have selected disorders of personality and behaviour (3% vs. 6%; *p* < 0.001) compared to males who were homeless.

## Discussion

Among hospitalizations for substance-related poisonings, males and younger adults were disproportionately represented among people experiencing homelessness, as compared to the housed population. Higher proportions of homelessness among men than among women have been previously reported.^2^,^18^ However, recent evidence suggests that many more women than men may be experiencing hidden homelessness, resulting in misclassification of housing status among females.^19^ The younger mean age of people experiencing homelessness hospitalized for substance-related poisoning observed in this study likely reflects the younger age of people who are homeless.^2^

We found that a higher proportion of substance-related poisoning hospitalizations among people experiencing homelessness were recorded as accidental rather than intentional self-harm, and that opioids and stimulants, notably fentanyl and its analogues and heroin, were most commonly involved in poisonings leading to hospitalizations. The large proportion of these poisonings being accidental is likely due to the increase in fentanyl and its analogues in the illicit (unregulated) drug supply. These substances have high potencies and are increasingly being combined with other controlled substances.^20^-^22^ The people who were hospitalized may not have known that the substance they were taking also contained fentanyl and/or its analogues, they may have combined substances to alleviate withdrawal symptoms or to enhance their experience,^23^ or the dose may have been higher than expected, leading to an accidental poisoning. 

Hospitalizations for substance-related poisonings among people experiencing homelessness were longer than for those who were housed, with total lengths of stay averaging 11 and 6.6 days, respectively. This finding may be explained by the higher rates of infectious diseases, chronic diseases and long-term physical health conditions prevalent among people who are homeless^24^,^25^ as well as higher rates of mental disorders, as observed in our study. Hospitalized individuals may have also received treatment for comorbidities, resulting in increasing lengths of stay. Further, the lengths of stay for alternate level of care may have differed between the two populations because hospitals may not have options for discharging patients experiencing homelessness.

Lastly, people who were homeless were more likely than those with housing to leave the hospital against medical advice or before being formally discharged by a health care professional. This finding is consistent with previous literature that people who are discharged against medical advice are more likely to be young, male and experiencing homelessness.^26^,^27^ Choi et al.^26^ found that people who are discharged against medical advice had higher rates of both readmission within 14 days and mortality within 12 months. This particular finding has important implications for clinical care settings looking for ways to decrease the number of patients leaving hospital against medical advice and thus reduce harms, mortality and associated costs and increase health care satisfaction.

***Strengths and limitations***

To our knowledge, this analysis is the first to examine characteristics of substance-related poisoning hospitalizations of people experiencing homelessness across Canada. The DAD includes inpatient acute hospitalization discharges from all provinces and territories except Quebec and therefore has substantial coverage of the population of interest in this study.

There are, however, limitations. First, this analysis only examined acute inpatient hospitalizations, and patterns of substance-related poisonings may vary across different health care settings. For example, individuals with less severe poisonings may be treated through emergency medical services or in the emergency department; not including these health care settings could lead to underestimating the overall prevalence of substance-related poisonings. Moreover, if the pattern of where people seek health care for such poisonings and who is admitted to hospital varies by housing status, these results may not adequately reflect true differences. 

Also not captured were data on people who died before being admitted to hospital, which potentially focused this analysis on less severe cases or instances where help was more readily available. 

The unit of analysis was hospital discharge and not at the person level or for entire episodes of care. People could have been readmitted multiple times during the study period, which would be counted as multiple hospitalizations. People with multiple admissions may have unique characteristics that are not presented in this study. 

Another limitation was that identification of homelessness status may have relied on self-reported information. It is possible that some patients may not have disclosed their homelessness status, or were unable to due to disability or death, which could have resulted in their being misclassified. Similarly, it was only possible to examine housing status as a binary term, as either experiencing homelessness, or not. More nuance is required by including unstable housing, poor housing quality, overcrowding or former homelessness to fully understand the impact of housing status. It is a relatively new requirement to record homelessness status on hospital discharge records and therefore a trend analysis was not possible. 

Identifying intention of the poisoning also relied on self-reported information, which can introduce bias if patients are unwilling or unable to disclose this information. Poisonings were classified as accidental unless other intentions were clearly documented, potentially leading to an overrepresentation of accidental poisonings. Throughout this analysis it was not possible to determine which poisonings were a result of pharmaceutical or illicit (or unregulated) opioids, or a combination of both, which hinders the ability to develop targeted interventions to reduce harms associated with substances from different sources. 

The estimates of recorded mental disorders did not reflect the overall prevalence of mental disorders among those hospitalized for substance-related poisonings; rather, the mental disorders that were recorded were relevant to the patient’s stay in hospital. 

Lastly, Canadian Armed Forces veterans are two to three times more likely to experience homelessness than the general population, and the absence of military status in these data hinders the ability to provide a comprehensive understanding of the relationships between military service, housing status and substance-related poisonings.^28^

***Implications***

The COVID-19 pandemic has widened health disparities, particularly among hard-to-reach populations.^2^-^4^ There has also been an increase in the number of people experiencing homelessness, as well as an increase in the number of substance-related poisonings across the country.^2^,^10^,^11^ Although we examined a pre-pandemic period, the results of this study could be used to support actions to reduce substance-related harms by strengthening public health and social infrastructure as people continue to experience the long-term impacts associated with the COVID-19 pandemic as well as other economic impacts.

These findings highlight the need for health care professionals, researchers and policy makers to better understand the intersection of homelessness, mental illness and substance-related harms. They can also inform sectors that interact with vulnerably housed individuals. In particular, these findings demonstrate how substance-related harms and care in hospital settings may differ for people with housing compared to people experiencing homelessness, as exhibited by the high proportion of substance-related poisoning hospitalizations that ended with leaving against medical advice. This difference in care may be due to a variety of factors, such as care not meeting the needs of this population or due to a lack of trust or stigma, and may warrant further investigation to reduce barriers to care for people who are homeless.

## Conclusion

Compared to people with housing, unhoused people hospitalized for a substance-related poisoning are more likely to be younger, male and with a recorded mental disorder. A higher proportion of substance-related poisoning hospitalizations of unhoused people were accidental and involving opioids and stimulants, particularly fentanyl and its analogues and heroin. Lastly, substance-related poisoning hospitalizations of unhoused people lasted longer and were more likely to end with leaving the hospital against medical advice. 

These findings emphasize the importance of acknowledging the intersectionality of mental illness, substance use and housing status when considering options to address substance-related harms. Future studies should aim to determine how care in hospital settings and other social services can optimize support, in order to prevent further substance-related harms.

## Acknowledgements

We thank the Canadian Institute for Health Information for collecting and providing the data used in this study, and Patrick Hunter and Nan Zhou from Infrastructure Canada for their input and support of this project. 

Some material from this report has been previously published by the Government of Canada (https://www.canada.ca/en/health-canada/services/opioids/hospitalizations-substance-related-poisonings-homelessness.html); permission was obtained to reprint it. All major contributors were contacted and agreed to this publication.

## Funding

This research did not receive any funding from agencies in the public, commercial or not-for-profit sectors. 

## Conflicts of interest

None to declare.

## Authors’ contributions and statement

RP: Investigation, data curation, methodology, formal analysis, writing – original draft.

RW: Investigation, data curation, methodology, formal analysis, writing – review & editing.

HO: Conceptualization, supervision, writing – review & editing.

VG: Conceptualization, supervision, validation, writing – review & editing.

Parts of this material are based on data and information compiled and provided by the Canadian Institute for Health Information (CIHI). However, the analyses, conclusions, opinions and statements expressed herein are those of the authors and not necessarily those of CIHI or of the Government of Canada.
